# Severe structural valve deterioration after TAVR with ACURATE Neo: report of two cases

**DOI:** 10.3389/fcvm.2023.1135496

**Published:** 2023-05-25

**Authors:** Thibault Schaeffer, Luca Koechlin, Raban Jeger, Gregor Leibundgut, Oliver Reuthebuch

**Affiliations:** ^1^ Department of Cardiac Surgery, University Hospital Basel, University of Basel, Basel, Switzerland; ^2^ Department of Cardiology, University Hospital Basel, University of Basel, Basel, Switzerland

**Keywords:** ACURATE Neo, TAVR explantation, structural valve deterioration (SVD), bioprosthesis failure, surgical aortic replacement

## Abstract

Structural valve deterioration (SVD) of transcatheter implanted aortic valve (TAVR) prostheses leading to prosthesis dysfunction is an uncommon yet increasingly described complication. Literature is scarce on specific mechanisms and clinical presentation of SVD after TAVR, notably on self-expanding valve ACURATE Neo. We report on two cases with severe bioprosthetic failure after ACURATE Neo implantation due to leaflet disruption, and we treated them with surgical aortic valve replacement. Based on the literature, we further discuss the incidence of SVD after TAVR, the durability of ACURATE NEO, and the modes of failure of biological valve prostheses.

## 1. Introduction

Structural valve deterioration (SVD) refers to intrinsic alterations of bioprosthetic heart valves such as leaflet fibrosis and/or calcification, leaflet tear, and pannus that eventually lead to their hemodynamic dysfunction. Bioprosthetic failure (BVF) defines any clinically expressive valve dysfunction related to SVD or other conditions, such as endocarditis and prosthetic valve thrombosis, that eventually requires valve reoperation or reintervention (1). The clinical manifestation of bioprosthetic failure varies according to the severity of valve dysfunction. Literature is scarce on specific mechanisms and clinical presentation of SVD after TAVR, notably on self-expanding valve ACURATE Neo (Boston Scientific, Ecublens, Switzerland). We herein report on two cases with distinct mechanisms of SVD after ACURATE Neo implantation leading to BVF.

## 2. Case description

### 2.1. Case 1

**Figure 1:**
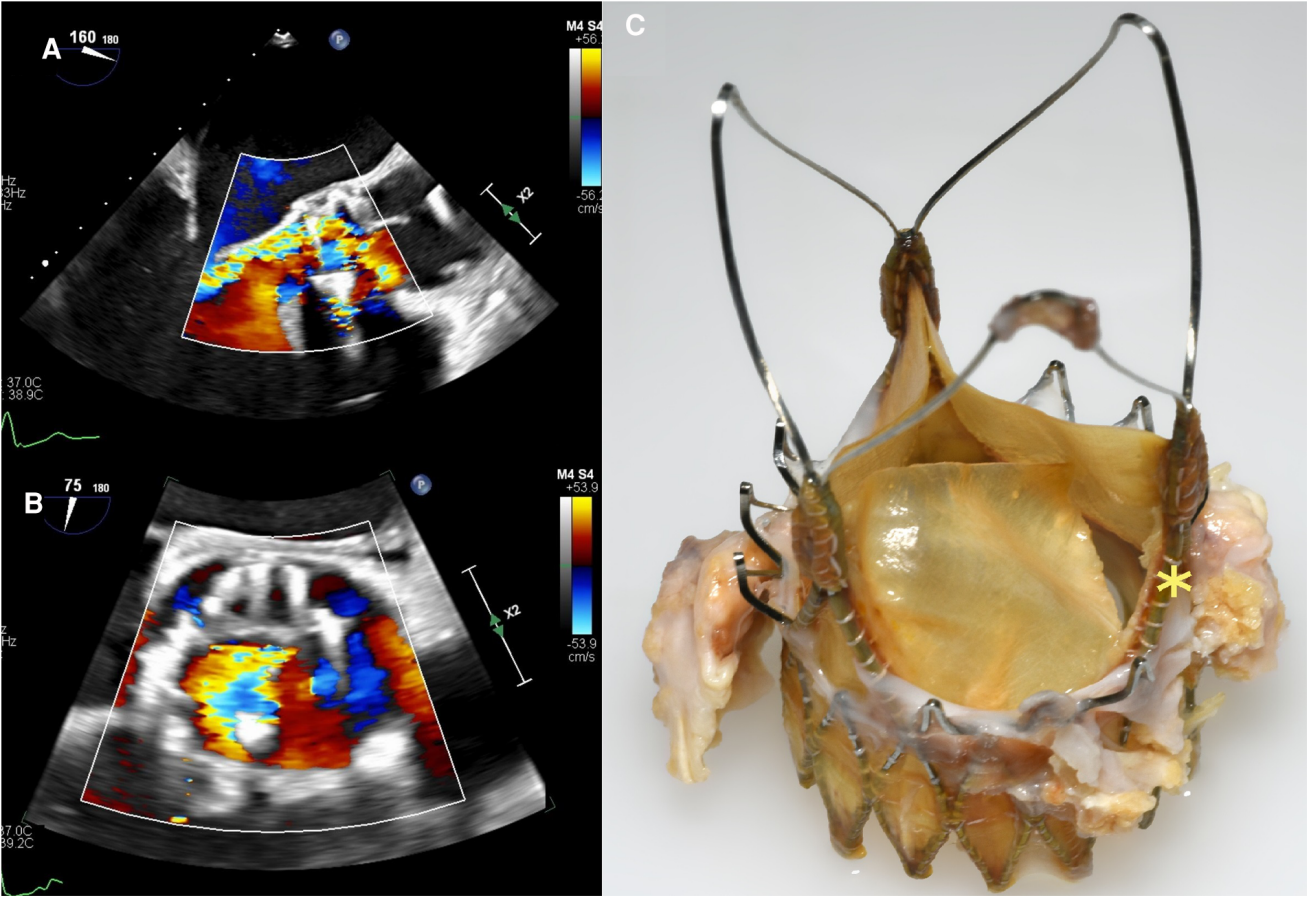
Case 1: transesophageal echocardiography three-chamber view with color-flow Doppler (**A**) and aortic valve-centered short-axis view with color-flow Doppler (**B**) showing an eccentric, severe intraprosthetic regurgitation. (**C**) Explanted ACURATE Neo prosthesis with leaflet disruption with its adjacent strut in the noncoronary position (yellow asterisk).

A 70-year-old man was implanted with ACURATE Neo (size M) for symptomatic, severe aortic valve stenosis. The procedure was unremarkable, and transthoracic echocardiography (TTE) at discharge showed a mean aortic transvalvular aortic gradient of 7 mmHg and trivial paravalvular regurgitation. Approximately 4 years (54 months) later, he presented to his regional hospital with rapidly progressive dyspnea. TTE revealed severe aortic valve regurgitation (AR). After transfer to our tertiary center, transesophageal echocardiography (TEE) demonstrated a well-seated TAVR prosthesis with severe intraprosthetic, eccentric AR (see Figures 1A,B). Transvalvular aortic gradients were slightly elevated (mean pressure gradient 18 mmHg). Blood cultures were negative. Considering the intermediate surgical risk of the patient, we opted for an emergent TAVR explant and surgical aortic valve replacement (SAVR). Intraoperative inspection of the prosthesis revealed a leaflet disruption with its adjacent strut in the noncoronary position (see Figure 1C). There were no signs of endocarditis. Extraction of the prosthesis was complicated due to extensive pannus arising from the heavily calcified native aortic valve. Due to subsequent lacerations of the aortic annulus, the aortomitral junction was reconstructed with a bovine pericardial patch, and a 23-mm stented bioprosthesis was implanted in the aortic position. The postoperative course was uneventful, and the patient was promptly discharged. After an internal review, the manufacturer was unable to identify the cause of prosthesis dysfunction. According to the manufacturer's archive, the referenced device had passed all tests during manufacturing and met all required specifications before approval for final distribution and sale.

### 2.2. Case 2

**Figure 2:**
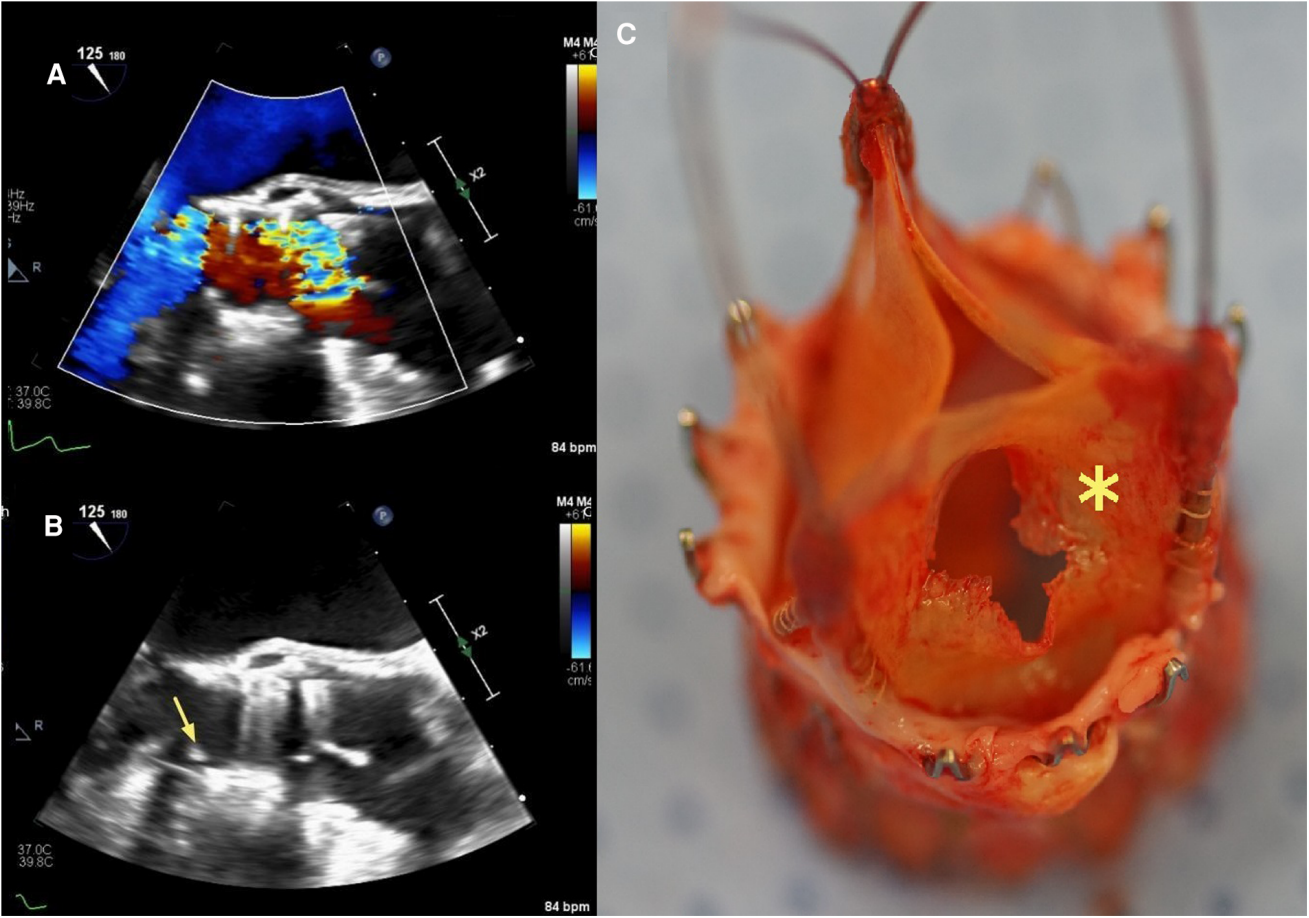
Case 2: transesophageal echocardiography three-chamber view with color-flow Doppler (**A**) and aortic valve-centered short-axis view with color-flow Doppler (**B**) showing severe intraprosthetic regurgitation and partial cusp prolapse (yellow arrow) on the right coronary side, respectively. (**C**) Explanted ACURATE Neo prosthesis with a central tear in the leaflet in the right-coronary position (yellow asterisk).

An 80-year-old woman was implanted with ACURATE Neo (size S) for symptomatic aortic valve stenosis. Moderate residual AR after valve deployment was immediately addressed with single balloon dilatation. TTE at discharge and at the 1-year routine control showed no significant regurgitation and low transaortic gradients. After barely 2 years (23 months), the patient complained of sudden dyspnea and medication-refractory elevated blood pressure. TTE performed by her cardiologist revealed a severe AR. TEE after admission at our center demonstrated a partial cusp prolapse on the right-coronary position associated with severe, eccentric AR (see Figures 2A,B). Blood cultures were negative. Given the intermediate surgical risk and to prevent coronary obstruction with limited coronary ostium height (13 mm on both sides), we opted for a TAVR explant and SAVR. Intraoperative inspection of the prosthesis revealed a central tear in the leaflet in the right-coronary position (see Figure 2C). There were no local signs of endocarditis. The extraction of the prosthesis was complicated due to an extensive pannus and endothelialization of the axial stabilization arches anchored in the aortic wall. After prosthesis removal, we implanted a 21-mm surgical bioprosthesis in the aortic position. The postoperative course was besides transitory acute-on-chronic renal failure uneventful, and the patient was discharged after 10 days. Cases summary as timeline is provided in Table 2.

## 3. Discussion

Data on specific mechanisms of SVD affecting TAVR prostheses, their incidence, and clinical presentation are limited.

The PARTNER-1 (Placement of Aortic Transcatheter Valves) trial (*n* = 1057) and CoreValve High-Risk Pivotal trial (*n* = 795) reported extremely low rates of severe SVD with 0% and 0.8% after 5 years for SAPIEN (Edwards Lifesciences, Irvine, CA, USA) and CoreValve (Medtronic, Minneapolis, MN, USA), respectively (2, 3). However, interpretation of these findings is limited due to the small number of survivors at 5 years, as predictable with typically elderly and frail patients included in these historical cohorts. With the introduction of standardized criteria for SVD, longer follow-up, and inclusion of younger, lower-risk patients in prospective TAVR trials in recent years, higher rates of SVD were reported. The NOTION (Nordic Aortic Valve Intervention) trial (*n* = 139), referring to the European Association of Percutaneous Cardiovascular Interventions (EAPCI), reported SVD risk after CoreValve implantation as high as 4.8% at 6 years (4). Analyzing data from the PARTNER-2A trial (*n* = 1438) and PARTNER-2/SAPIEN-3 Intermediate-Risk registry (*n* = 891) and referring to the Valve Academic Research Consortium 3 definitions, Pibarot et al*.* reported SVD risks of 3.9% and 9.5% at 5 years after implantation of Edwards SAPIEN 3 and Edwards SAPIEN XT, respectively (5). However, the rates of BVF were rather low (1.1% and 3.7% for Edwards SAPIEN 3 and Edwards SAPIEN XT, respectively). Also, large European retrospective cohort studies on multiple TAVR devices reported 5-year rates of SVD ranging from 2.5% to 4.2% and low rates of SVD-related BVF according to the EAPCI guidelines (6, 7). The heterogeneity of the above-mentioned rates of SVD makes their interpretation difficult and can be explained by at least two factors: first, the underestimation of the incidence of SVD given the substantial competitive risk of death in cohorts with a high mortality on short term (e.g., PARTNER-1), and second, the variability of SVD criteria according to the definitions used. An overview of SVD rates reported by the major historical randomized control trials and retrospective cohort studies focusing on TAVR durability is given in Table 1.

**Table 1:** Overview of SVD rates reported by the major historical randomized control trials and retrospective cohort studies focusing on TAVR durability.

Author	Year	Trial/referred trial/register	Type of study	*n*	TAVR prosthesis	Follow-up (years)	SVD^a^ (%)	BVF (%)	All-cause mortality (%)	Definition of SVD
Kapadia et al.	2015	PARTNER-1	RCT	1,057	SAPIEN	5	0.00	*NA*	71.80	*NA*
Gleason et al.	2018	CoreValve High-Risk Pivotal	RCT	391	CoreValve	5	0.8	*NA*	55.00	EAPCI
Søndergaard et al.	2019	NOTION	RCT	139	CoreValve	6	4.8	6.70%	42.50	EAPCI
Didier et al.	2018	FRANCE-2	Retrospective	4,201	CoreValve SAPIEN SAPIEN XT	5	2.50	NA	60.80	EAPCI
Durand et al.	2019	NA	Retrospective	1,403	SAPIEN SAPIEN XT CoreValve Jena	5	4.2	1.90	69.00	EAPCI
Pibarot et al.	2020	PARTNER-2A PARTNER-2/SAPIEN-3	Retrospective	774	SAPIEN 3	5	3.90	1.1^b^	NA	VARC-3
				891	SAPIEN XT		9.50	3.7^b^	NA	VARC-3
Testa et al.	2020	NA	Retrospective	990	CoreValve	8	1.6	2.50	78.30	EAPCI
Tamburino et al.	2020	SCOPE-2	RCT	398	ACURATE Neo	1	10.00	0.25^b^	13.00	VARC-2
				398	CoreValve Evolut		14.00	1^b^	9.00	VARC-2
Siquiera et al.	2021	NA	Retrospective	104	ACURATE Neo	3	1.00	1^b^	20.70	VARC-2

TAVR, transcatheter aortic valve replacement; SVD, structural valve deterioration; BVF, bioprosthetic valve failure; RCT, randomized controlled trial; VARC, Valve Academic Research Consortium; EAPCI, European Association of Percutaneous Cardiovascular Interventions; NA, not available.

^a^ For study referring to the EAPCI definition, only severe SVD is reported.

^b^ Specifically, SVD-related bioprosthetic failure.

**Table 2:** Timeline.

Case 1		Case 2	
Time	Event	Time	Event
*T*_0_	TAVR with ACURATE Neo	*T*_0_	TAVR with ACURATE Neo
*T*_1 _= T_0_ + 54 months	Hospital admission for rapidly progressive dyspnea/pulmonary edema	*T*_1 _= *T*_0_ + 23 months	Cardiological workup for acute dyspnea and medication-refractory elevated blood pressure
*T*_1_ + 7 days	TEE reveals eccentric, severe intraprosthetic AR	*T*_1_ + 15 days	TEE reveals severe intraprosthetic AR and partial cusp prolapse
*T*_1_ + 9 days	Surgical explant of ACURATE Neo, SAVR	*T*_1_ + 19 days	Surgical explant of ACURATE Neo, SAVR
*T*_1_ + 22 days	Hospital discharge	*T*_1_ + 29 days	Hospital discharge

TAVR, transcathter aortic valve replacement; TTE, transthoracic echocardiography; AR, aortic regurgitation; SAVR: surgical valve replacement.

When looking at surgical registries of SAVR after TAVR (*n* = 123 and *n* = 46), SVD accounted for 10%–15% of indications for TAVR explant in two recent retrospective studies (8, 9).

Since ACURATE Neo is a more recent and less implanted model of the TAVR era, less information is available about long-term outcomes and specific failure mechanisms of this device.

In the SCOPE-2 (Safety and Efficacy Comparison of Two TAVR Systems in a Prospective Randomized Evaluation 2) trial (*n* = 796), ACURATE Neo was challenged with its self-expandable concurrent Medtronic CoreValve Evolut (10). The authors observed more frequent cardiac deaths at 1 year (8.4% vs. 3.9%; *p *= 0.01), more frequent severe aortic regurgitation in the short term (10% vs. 3%, *p *= 0.002), and more frequent structural valve deterioration in the short term (14% vs. 6%, *p *= 0.004) with ACURATE Neo. However, structural valve deterioration was no more statistically different after 1 year (10% vs. 14%, *p *= 0.25) and the valve-related dysfunction requiring repeat procedure was similarly low in both devices (1%, *p *= 0.99). Reporting on mid- and long-term results after ACURATE Neo implantation (*n* = 104), Siquiera et al*.* mentioned a single case of SVD-related failure addressed with a *valve-in-valve* procedure (11). The authors concluded with overall reassuring mid- to long-term outcomes with this device. Findings of the SCOPE-2 trial at 1 year and those of Siquiera et al. are summarized in Table 1. In our institution, we observed a rate of SVD-related BVF of 0.84%, accounting for the two present cases over the past 5 years, which was similar to the previously mentioned studies. Finally, reviewing the international EXPLANT-TAVR registry (*n* = 269), Bapat et al*.* noticed six surgical explants of ACURATE Neo (4.5% of all self-/mechanically expandable devices), but neither the causes for explant nor the durability of the failed prostheses was specified (12).

As the number of TAVR in even younger patients increases, the need to manage late complications with TAVR will increase. The number of former implanted and available TAVR models is also increasing. As such, a better understanding of the specific mechanisms of SVD affecting TAVR prostheses is mandatory. Our two cases contribute to increasing knowledge in this area by illustrating two close but specific modes of SVD leading to acute bioprosthetic failure of the ACURATE Neo. A macroscopic examination of the valves revealed no signs of active endocarditis, and the blood cultures were negative. Eubacterial PCR from leaflet samples was not performed to exclude previous endocarditis. Leaflet tear/disruption is a rare yet well-documented mechanism of SVD of surgical bioprostheses, especially those with externally mounted leaflets (13–16). Leaflet disruption in aortic bioprosthesis occurs abruptly, and the affected patient typically presents with acute, clinically poorly tolerated aortic regurgitation, as in the two present cases. In the absence of endocarditis criteria, we assumed that the rupture of the leaflet was due to mechanical fatigue by an analogous mechanism to that affecting surgical bioprostheses. To the best of our knowledge, this issue with ACURATE Neo has never been reported.

As for therapy, we opted for TAVR explant and SAVR. Our strategy was driven in both cases by the limited experience reported with *valve-in-valve* procedures in failed ACURATE Neo, in the second case by the rather shallow aortic root with subsequent risk of coronary obstruction. Indeed, recent works highlighted the increased risk of coronary obstruction following redo-TAVR with high-profile index bioprosthetic valves such as ACURATE Neo (17). Further studies are warranted to define the best approach to failed TAVR prostheses with respect to the index model, mode of failure, and patient´s anatomy.

## Data Availability

The data analyzed in this study is subject to the following licenses/restrictions: Data are available on reasonable request to the corresponding author. Requests to access these datasets should be directed to oliver.reuthebuch@usb.ch.
